# Chromosomal Microarray Analysis in Critically Ill Neonates and Children: Diagnostic Yield and Clinical Utility

**DOI:** 10.3390/life16061034

**Published:** 2026-06-22

**Authors:** Joshua Meyer, Emily Hershman, Ananditha Sivakumaran, Vinisha Venugopal, Derek Neilson, Theresa A. Grebe, Theru A. Sivakumaran

**Affiliations:** 1 School of Medicine, Creighton University, Phoenix, AZ 85012, USA; 2 Division of Pathology and Laboratory Medicine, Phoenix Children’s Hospital, Phoenix, AZ 85016, USA; 3 Sri Ramachandra Medical College & Research Institute, Chennai 600116, India; 4 Division of Genetics and Metabolism, Phoenix Children’s Hospital, Phoenix, AZ 85016, USA; 5 Department of Child Health, University of Arizona College of Medicine-Phoenix, Phoenix, AZ 85004, USA; 6 Department of Pathology, University of Arizona College of Medicine-Phoenix, Phoenix, AZ 85004, USA; 7 Department of Pathology, Creighton University School of Medicine, Phoenix, AZ 85012, USA

**Keywords:** chromosomal microarray analysis, copy number variants, diagnostic yield, critically ill children, congenital anomalies, congenital heart defects, developmental delay, genomic testing, uniparental disomy, absence of heterozygosity

## Abstract

Chromosomal microarray analysis (CMA) is widely used to detect chromosomal aneuploidies and copy number variants (CNVs) in pediatric patients with congenital anomalies or developmental concerns. However, its diagnostic utility in critically ill neonates and children admitted to intensive care units (ICUs) remains undercharacterized. We conducted a retrospective review of 679 patients admitted to the neonatal, pediatric, or cardiovascular intensive care units (NICU, PICU, CVICU) at Phoenix Children’s Hospital between 2019 and 2024 who underwent CMA. Demographic data, clinical indications, and CMA results were extracted from electronic medical records to assess diagnostic yield and variant patterns. CMA identified a clinically relevant finding in 102 of 679 patients, resulting in an overall diagnostic yield of 15.0% (95% CI: 12.3–17.7%). Clinically relevant findings included pathogenic (P) variants (n = 88), likely pathogenic (LP) variants (n = 12), and large regions of absence of heterozygosity (AOH) consistent with uniparental disomy (UPD) (n = 2). A variant of uncertain significance (VUS) was detected in 139 patients (20.5%). Among the pathogenic and likely pathogenic variants, CMA identified recurrent CNVs (n = 49), nonrecurrent CNVs (n = 17), aneuploidies (n = 22), and patients with two pathogenic or likely pathogenic CNVs (n = 10). Diagnostic yields of 48.4% (95% CI: 38.5–58.4%) and 8.4% (95% CI: 6.0–11.5%) were observed in patients with single or multiple congenital anomalies including a congenital heart defect (CA + CHD), and in patients with an isolated CHD, respectively. CMA demonstrates significant diagnostic value in critically ill neonates and children, particularly among those with multisystem congenital anomalies. These findings support the routine integration of CMA in genomic evaluation protocols for ICU populations to guide diagnosis, management, and counseling.

## 1. Introduction

Chromosomal microarray analysis (CMA) is a cornerstone of clinical genetic testing, particularly for detecting chromosomal aneuploidies and submicroscopic copy number variants (CNVs) in individuals with unexplained developmental delay (DD), intellectual disability (ID), congenital anomalies (CA), and autism spectrum disorder (ASD) [1,2,3]. Since 2010, CMA has been recognized as a first-tier cytogenetic test in both the United States and Europe, due to its superior diagnostic yield compared with conventional karyotyping [3,4,5].

In neonatal and pediatric intensive care units (NICU, PICU, and CVICU), where patients often present with complex, multisystem anomalies, a rapid genetic diagnosis can meaningfully inform and guide clinical care [6]. Recent implementation of rapid ES/GS programs in the NICU setting, including initiatives such as NICUSeq, BabySeq, and Project Baby Bear, has further expanded the role of genomic testing in critically ill infants by demonstrating improved diagnostic yield, reduced time to diagnosis, and potential impacts on clinical management and healthcare utilization [7,8,9,10]. These programs highlight the increasing integration of rapid sequencing technologies into NICU workflows, particularly for patients with suspected monogenic disorders or unexplained multisystem disease.

Although professional societies such as the American Academy of Pediatrics (AAP) and American College of Medical Genetics and Genomics (ACMG) recommend exome sequencing (ES) or genome sequencing (GS) as first-tier testing for critically ill patients, access to these modalities remains limited across many inpatient settings [11]. A survey of level IV NICUs found that, although ES/GS was universally available, access was restricted at 81% of centers, most commonly requiring specialist approval [12]. Additional barriers include insurance limitations, institutional variability in test availability, insufficient genetics workforce capacity, and concerns surrounding informed consent processes [12,13,14]. As a result, CMA continues to serve as a practical first-tier diagnostic modality in many inpatient settings, particularly where timely access to ES/GS remains constrained by these institutional and systemic barriers. In addition, the latest AAP guidelines continue to recommend CMA as a first-tier agnostic evaluation along with sequential or concurrent ES [11].

Despite continued utilization in intensive care populations, the diagnostic yield and clinical impact of CMA in critically ill neonates and children remains incompletely characterized in the literature.

This retrospective study examines the diagnostic yield and clinical significance of CMA in a cohort of 679 patients admitted to intensive care units at Phoenix Children’s Hospital (PCH) between 2019 and 2024. By examining CMA results alongside patient characteristics and testing indications, this study aims to (1) quantify the diagnostic yield of CMA, (2) describe the spectrum of pathogenic and likely pathogenic CNVs identified, and (3) assess the clinical relevance of these findings in the context of intensive care. These findings contribute to the growing evidence base supporting utilization of CMA in critically ill pediatric populations and help inform best practices for integrating genomic testing into ICU workflows.

## 2. Materials and Methods

### 2.1. Patient Samples

This retrospective study evaluated patients who underwent constitutional chromosomal microarray analysis (CMA) testing at the Phoenix Children’s Clinical Genomics Laboratory from 2019 to 2024 while admitted to the neonatal intensive care unit (NICU), pediatric intensive care unit (PICU), or cardiovascular intensive care unit (CVICU). In accordance with ACMG recommendations during the study period, CMA was typically ordered for critically ill patients with CA, DD, dysmorphic features, congenital heart defects, or other clinical findings raising suspicion for an underlying chromosomal abnormality or genomic syndrome. A total of 679 unrelated patients were included. When clinically indicated, confirmatory or follow-up testing was recommended by the laboratory and ordered at the discretion of the treating provider. However, not all findings underwent confirmatory evaluation due to factors including loss to follow-up, insurance limitations, clinical circumstances, or provider preference. This study was granted a waiver of consent by the Phoenix Children’s Institutional Review Board.

### 2.2. Microarray Analysis

Genomic DNA was isolated from peripheral blood, or buccal swab samples using the QIAsymphony DSP DNA Midi Kit on the QIAsymphony instrument (Qiagen Inc., Germantown, MD, USA). DNA samples were digested, amplified, labeled, and hybridized to the CytoScan HD microarray following the manufacturer’s protocol (Thermo Fisher Scientific, Inc., Waltham, MA, USA). Raw data were analyzed using Chromosomal Analysis Suite (ChAS) software versions 4.0 and 4.3 to identify copy-number variants (CNVs) and regions of homozygosity (ROH) ≥ 5 Mb. Genomic coordinates were based on the human genome reference sequence GRCh37/hg19. CNVs were classified as pathogenic (P), likely pathogenic (LP), variants of uncertain significance (VUS), likely benign, or benign following American College of Medical Genetics and Genomics recommendations [15].

### 2.3. Electronic Medical Record Review

Electronic medical records for all 679 patients were reviewed to obtain detailed phenotypic information and demographic characteristics, as well as results from ancillary laboratory studies.

### 2.4. Data and Statistical Analysis

Descriptive statistics summarized patient characteristics, including age at testing and clinical indications. Diagnostic yield was defined as the proportion of patients with clinically significant findings, including pathogenic/likely pathogenic (P/LP) variants and clinically significant AOH/UPD findings, among all tested patients within each clinical indication category. Clinical indication groups included congenital heart defects with or without other findings (CHD ± others), isolated CHD, congenital heart defects with central nervous system abnormalities or developmental delay (CHD + CNS/DD), CHD including single or multiple other congenital anomalies (CHD + CA), single or multiple congenital anomalies without CHD (CA—CHD), CNS/DD (isolated), and other. The “Other” category comprised lower-frequency indications, including respiratory, musculoskeletal, and other miscellaneous clinical presentations. Diagnostic yield was estimated along with the corresponding 95% confidence interval (CI) using the Wilson method.

Associations between diagnostic yield and demographic or clinical variables were evaluated using Fisher’s exact test. For comparisons involving more than two categories (e.g., race and grouped clinical indications), Fisher’s exact test with Monte Carlo simulation was performed to estimate global *p*-values. These analyses were designed to assess overall associations between clinical categories and diagnostic outcomes rather than differences between individual subgroup pairs; therefore, post hoc pairwise comparisons and multiple-comparison corrections were not performed. This approach was selected because several clinical indication groups had limited sample sizes, which could reduce the reliability and interpretability of multiple pairwise comparisons. All statistical analyses were performed in R 4.5.0, and two-sided *p*-values < 0.05 were considered statistically significant.

## 3. Results

### 3.1. Patient Cohort

**Figure 1:**
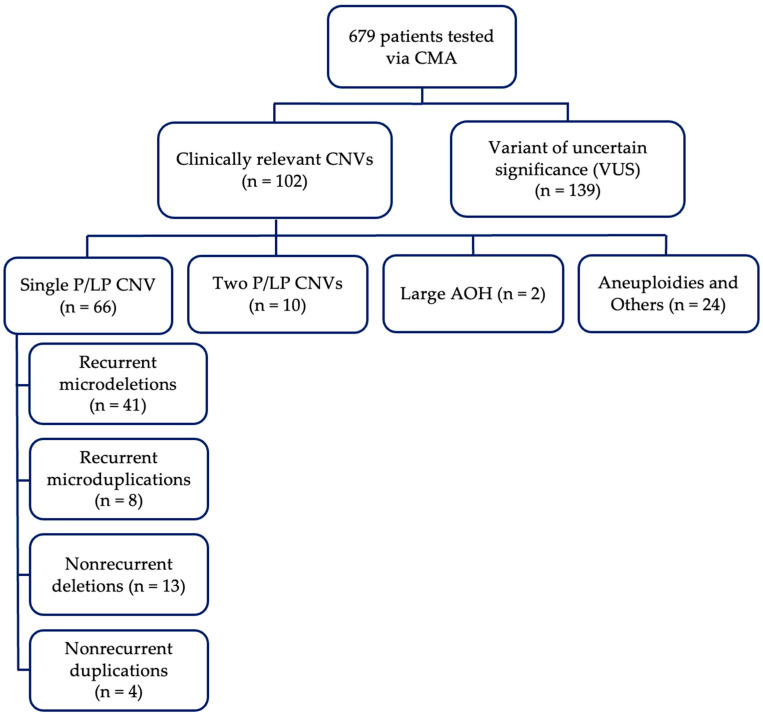
Flowchart of CMA findings in critically ill patients.

A total of 679 patients admitted to the NICU, PICU, or CVICU at Phoenix Children’s Hospital underwent chromosomal microarray analysis (CMA) between 2019 and 2024. Of those tested, 45.9% (n = 312) were female and 54.1% (n = 367) were male. The median age at testing was 7 days (IQR: 4–14 days; range: 0–15 years). More than 85% (578/679) were tested before one month of age; 11.8% (80/679) between 1 and 6 months, 1.2% (8/679) between 6 and 12 months, and 1.9% (13/679) were older than one year at testing. Of these 679 patients, 102 (15.0%) had a clinically relevant copy number variant (CNV), and 139 (20.5%) had a variant of uncertain significance (VUS). Among the 102 clinically relevant CNVs, 88 were classified as pathogenic (P), 12 as likely pathogenic (LP), and 2 demonstrated large regions of absence of heterozygosity (AOH) consistent with uniparental disomy (UPD). A summary of CMA findings for the cohort is shown in Figure 1.

**Figure 2:**
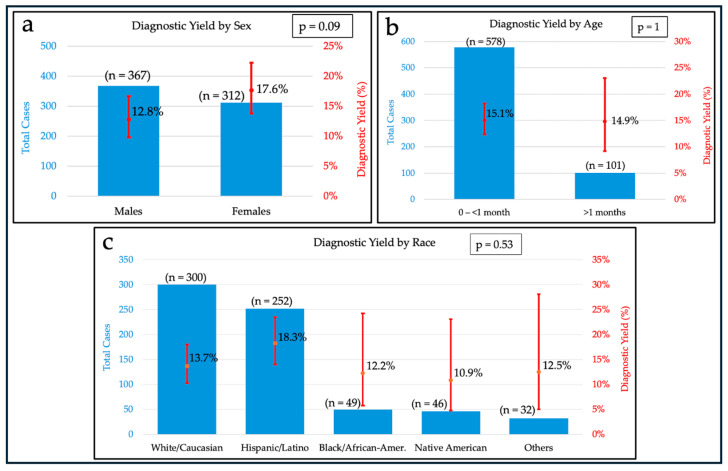
Diagnostic rate by sex, age, and race among patients who underwent CMA. (**a**) Distribution of total cases and diagnostic rates by sex. (**b**) Distribution of total cases and diagnostic rates by age group. (**c**) Distribution of total cases and diagnostic rates by race. Total case numbers are shown as blue bars, and diagnostic rates are represented by red points.

A breakdown of sex, age, and race distribution, along with the diagnostic rate by subgroup is provided in Figure 2. The diagnostic rate represents the proportion of patients within each subgroup who were found to have a clinically relevant chromosomal abnormality (i.e., pathogenic or likely pathogenic CNV) on CMA testing.

Diagnostic yield did not differ significantly by age, sex, or race. The yield was comparable between patients tested at <1 month (15.1%, 87/578; 95% CI 12.4–18.2%) and those tested at >1 month (14.9%, 15/101; 95% CI 9.2–23.1%), with no evidence of an association (Fisher exact *p* = 1.0). Similarly, although females demonstrated a higher diagnostic yield (17.6%, 55/312; 95% CI 13.8–22.2%) compared to males (12.8%, 47/367; 95% CI 9.8–16.6%), this difference did not reach statistical significance (*p* = 0.085). Across racial groups, diagnostic yield ranged from 10.9% to 18.3% (Black/African American: 12.2%, 6/49; Hispanic/Latino: 18.3%, 46/252; Native American: 10.9%, 5/46; Other: 12.5%, 4/32; White/Caucasian: 13.7%, 41/300), with no statistically significant differences observed (Fisher exact test with Monte Carlo simulation, *p* = 0.53). Racial categories with low sample sizes (Asian, Unknown, Hawaiian/Pacific Islander, and Other) were combined into a single group to ensure adequate counts for analysis. Collectively, these findings indicate that demographic variables were not significantly associated with diagnostic yield in this cohort.

### 3.2. Overview of Clinically Significant CNVs by Genetic Subtype

To facilitate a comprehensive characterization of patients with clinically significant CMA findings, individuals with pathogenic or likely pathogenic CNVs were systematically categorized according to genetic subtype, as presented in Figure 1. Four independent groups were observed: those with a single pathogenic or likely pathogenic CNV (n = 66; 64.7%), those with two identified pathogenic or likely pathogenic CNVs (n = 10; 9.8%), those with large regions of AOH consistent with possible UPD (n = 2; 2.0%), and those with aneuploidies along with sex chromosome-related disorders of sexual development (n = 24, 23.5%). Single pathogenic or likely pathogenic CNVs were further subdivided into the following categories: recurrent microdeletion syndromes, recurrent microduplication syndromes, nonrecurrent microdeletions, and nonrecurrent microduplications. Recurrent CNVs are defined as deletions or duplications that occur repeatedly in unrelated individuals at approximately the same chromosomal location, usually with similar breakpoints. Nonrecurrent CNVs are defined as deletions or duplications that occur at variable genomic regions and/or have unique breakpoints that differ between individuals. A complete breakdown of clinically relevant CNVs by genetic subtype and their relative proportions is provided in Table 1.

### 3.3. Recurrent CNVs Among Affected Patients

Of the 66 patients with a single CNV, 41 (62.1%) were diagnosed with recurrent microdeletion syndromes. Deletions involving 22q11.21 were the most common among patients with single CNV, identified in 40.9% (27/66), followed by 7q11.23 in 6.1% (4/66), 15q11.2 in 6.1% (4/66), 16p11.2 in 4.5% (3/66), 1q21.1 in 3.0% (2/66), and 17q12 in 1.5% (1/66). Notably, one patient (CMRES-2183) was identified with two independent pathogenic CNVs, including a 22q11.21 deletion, further supporting the recurrent occurrence of this microdeletion within the ICU cohort. In addition, eight patients (12.1%) were diagnosed with recurrent microduplication syndromes, most commonly involving 22q11.2 in 7.6% (5/66) of cases. Duplications at 16p11.2, 16p13.11, and 17q12 each were observed in 1.5% (1/66) of cases.

### 3.4. Nonrecurrent CNVs Among Affected Patients

Pathogenic and likely pathogenic nonrecurrent CNVs were identified across multiple chromosomal regions and varied substantially in size, gene content, and known clinical associations. Several CNVs overlapped, in whole or in part, genomic intervals that have been reported in association with established syndromic disorders or recognizable genotype–phenotype patterns. These included deletions involving 1p36.32, partially overlapping the distal 1p36 critical region associated with 1p36 deletion syndrome; 2q37.3, overlapping the region associated with 2q37 deletion syndrome; 9p24.3–p22.2, consistent with reported terminal 9p deletion phenotypes; 9q34.3, involving a deletion including *NOTCH1*; 13q12.3–q21.33, consistent with 13q deletion syndrome; and 22q13.2–q13.33, consistent with Phelan-McDermid syndrome. A deletion involving 14q32.2–q32.33 encompassed an imprinted region associated with Kagami–Ogata and Temple syndromes depending on parent-of-origin mechanisms. A smaller deletion at 12q15–q21.1, including *CNOT2*, overlapped deletions previously reported as 12q15 microdeletion syndrome in the literature. Additional nonrecurrent CNVs did not correspond to widely recognized recurrent genomic disorder intervals but have been reported in association with neurodevelopmental delay and congenital anomalies. These included interstitial deletions at 1q25.1–q32.1, 3p14.1–p12.2, 4q31.21–q31.22, and 18p11.32–p11.31, as well as duplications involving 2p22.1–p15, 8q12.1–q21.11, 8q21.2–q24.13, and 9p24.3–p21.3. A detailed summary of these CNVs, associated gene content, and phenotypes from the most recent chart review is provided in Table 2. Given the retrospective nature of this study and the variable expressivity associated with many CNVs, several reported clinical findings should be interpreted cautiously, as definitive genotype–phenotype relationships cannot be established in all cases, and some observed features may be incidental or multifactorial.

### 3.5. Two Pathogenic or Likely Pathogenic CNVs

Among the 102 patients with pathogenic or likely pathogenic CNVs or with AOH consistent with UPD, CMA identified two pathogenic or likely pathogenic CNVs in 10 patients (9.8%). Importantly, these CNVs were not uniformly interpreted as independent events. In at least half of these patients, the CNV patterns, typically consisting of a terminal deletion on one chromosome and a terminal duplication on another, were consistent with an underlying unbalanced translocation, raising the possibility of a balanced translocation in one of the parents. These cases included CMRES-1947, CMRES-1388, CMRES-0763, CMRES-1864, and CMRES-1150. Conventional chromosome analysis was recommended in these cases to further evaluate for potential underlying structural rearrangements.

In one patient (CMRES-0834), two CNVs involving chromosome 8 were identified, including an approximately 6.9 Mb terminal deletion at 8p23.3p23.1 and an approximately 31.2 Mb adjacent duplication at 8p23.1p11.1. This pattern of a terminal deletion with a more proximal duplication is characteristic of an inverted duplication with a terminal deletion of 8p (invdupdel [8p]), a recognized complex rearrangement mechanism associated with developmental delay, congenital anomalies, and other multisystem features. These findings were therefore interpreted as consistent with a possible invdupdel (8p) rearrangement rather than two independent pathogenic events.

In another case (CMRES-1392), CMA identified a duplication involving 17q that was suspected to represent a supernumerary marker chromosome, such as a ring chromosome 17, based on the size and distribution of duplicated material. An additional approximately 11.8 Mb duplication at 2q33.1q34, encompassing multiple genes including *BMPR2*, *CPS1*, *SUMO1*, *IDH1*, *CASP8*, and *CASP10*, was also detected. Partial duplications of 2q33 have been rarely reported but are associated with developmental delay and minor anomalies. Because these findings involved different chromosomes and lacked a shared structural mechanism, the 2q duplication was considered a possible independent pathogenic event in addition to the suspected marker chromosome.

Only cases with interstitial CNVs without evidence of a shared structural mechanism were considered likely to represent independent pathogenic events. These included CMRES-2183 and CMRES-3688; CMRES-1392 also met criteria for a possible independent pathogenic CNV in addition to the suspected marker chromosome.

For CMRES-2469, two adjacent CNVs involving 5p were identified, including an approximately 5.6 Mb deletion at 5p15.33p15.32 and a larger approximately 10.3 Mb mosaic deletion at 5p15.32p15.1, present in about 70% of cells. The mosaic loss was directly adjacent to the nonmosaic terminal deletion, raising the possibility that these findings represent a single complex or evolving structural event rather than two independent pathogenic CNVs. Both regions overlap the well-described 5p deletion region associated with Cri-du-chat syndrome. In addition, mosaic copy-neutral absence of heterozygosity of approximately 48 Mb involving the entire short arm of chromosome 11 (11p), encompassing the Beckwith–Wiedemann/Russell–Silver syndrome critical region, was identified, consistent with possible mosaic uniparental disomy; however, confirmatory UPD testing was not performed. Due to the lack of confirmatory testing, these results should be interpreted as provisional.

Overall, these findings indicate that the presence of two pathogenic or likely pathogenic CNVs in a single individual frequently reflects an underlying chromosomal rearrangement rather than multiple independent mutational events. This distinction is clinically relevant, as rearrangement-mediated CNVs have implications for parental carrier status and recurrence risk, whereas truly independent interstitial CNVs suggest separate pathogenic mechanisms. A detailed summary of these cases is provided in Table 3.

### 3.6. Absence of Heterozygosity Consistent with UPD

Of the 102 total patients with clinically significant CMA findings, two patients (2.0%) demonstrated large contiguous regions of absence of heterozygosity (AOH) involving a single chromosome, suggestive of possible uniparental disomy (UPD). CMA did not identify any pathogenic or likely pathogenic copy number variants in either case.

The first case (CMRES-0544) was a 5-day-old male with prenatal polyhydramnios and postnatal findings of ear malformations, musculoskeletal contractures, vertebral anomalies including scoliosis, and clinical features overlapping Jeune syndrome. CMA identified two large AOH intervals on the long arm of chromosome 14, spanning 14q11.2–q12 (~10.77 Mb) and 14q24.2–q32.33 (~34.72 Mb), raising concern for UPD(14). This patient has been comprehensively characterized clinically and genetically in a separate publication [16].

The second case (CMRES-3315) involved a 12-week-old premature male who presented with sepsis, respiratory distress, global hypotonia, lethargy, and limited behavioral responsiveness. CMA identified two large AOH segments on chromosome 15: a ~13.64 Mb interval spanning 15q11.2–q14, including the Prader–Willi/Angelman syndrome (PWS/AS) critical region (BP1–BP3), and a second ~26.28 Mb interval within 15q23–q26.2. Follow-up testing confirmed maternal UPD(15), establishing a diagnosis of Prader–Willi syndrome.

### 3.7. Aneuploidies

Of the 102 total patients with clinically significant CMA findings, aneuploidies were identified in 22 (21.6%), including Trisomy 21 in 16 (15.7%), Trisomy 18 in 3 (2.9%), Trisomy 13 in 1 (1.0%), and Monosomy X in 2 (2.0%). Follow-up chromosome analysis was not routinely available for patients with aneuploidies identified by CMA. Therefore, association between free trisomy, Robertsonian translocations-associated trisomy, and other chromosomal mechanism could not be consistently assessed. In addition to whole-chromosome aneuploidies, two cases demonstrated sex chromosome abnormalities: One patient exhibited 45,X/46,XY mosaicism, indicating the presence of both monosomic (45,X) and typical male (46,XY) cell lines, while another patient was a phenotypic female with a 46,XY karyotype. Both patients with sex chromosome abnormalities possessed congenital heart defects in addition to other congenital anomalies which prompted ICU admission and subsequent CMA.

### 3.8. Diagnostic Yield by Clinical Indication

To enable clinically meaningful interpretation of genotype–phenotype relationships and to evaluate diagnostic yield across presenting phenotypes, patients with clinically relevant CNVs were categorized according to the primary clinical indication at the time of referral into the following groups: congenital heart defects with or without other findings (CHD ± others), isolated CHD, congenital heart defects with central nervous system abnormalities or developmental delay (CHD + CNS/DD), CHD including single or multiple other congenital anomalies (CHD + CA), single or multiple congenital anomalies without CHD (CA—CHD), CNS/DD (isolated), and other. The CNS/DD group contained patients possessing structural brain malformations, abnormal electroencephalograms (EEG), or both. Conditions categorized as “other” included conditions that could not be reasonably assigned to the predefined diagnostic categories, including but not limited to cardiac abnormalities not associated with structural malformations (e.g., tachycardia) and perinatal conditions (e.g., intrauterine growth restriction). The categories of isolated CHD, CHD + CNS/DD, and CHD + CA were all subgroups of the CHD ± others category.

The most common clinical indications for which CMA was ordered were CHD ± others (73.8%, n = 501) and CA—CHD (17.2%, n = 117). Overall, the distribution of clinically significant CNVs across indication categories generally mirrored the relative frequency with which CMA was ordered in the ICU cohort; however, several notable exceptions were observed. For example, although CHD + CA accounted for only 13.7% (n = 93) of all CMA orders, this group represented 44.1% (n = 45) of the 102 clinically significant CNVs identified, yielding a detection rate of 48.4%. A similar trend was observed for the CHD + CNS/DD category, which accounted for only 1.9% (n = 13) of cases in which CMA was ordered, yet represented 6.8% (n = 7) of the clinically significant CNVs identified, yielding a diagnostic rate of 53.8% (95% CI: 29.1–76.8%). However, a limitation of this study was the small sample size within this group and thus additional studies with larger sample sizes are needed to confirm this trend observed in the CHD + CNS/DD subgroup. In contrast to the CHD + CA and CHD + CNS/DD categories, isolated CHD represented the most common indication for CMA, comprising 58.2% (n = 395) of cases while only 32.4% (n = 33) of clinically relevant CNVs were identified in this group, resulting in a diagnostic yield of 8.4% (95% CI: 5.6–11.1%). A complete breakdown of diagnostic yields across these clinical categories is provided in Table 4.

Diagnostic yield varied considerably across clinical indication categories. The highest detection rate was observed in patients with CHD + CNS/DD (53.8%); however, this estimate is exploratory and requires further confirmation in larger cohorts. Higher detection rates were also observed in the CHD + CA (48.4%) group. In contrast, substantially lower yields were observed in isolated CHD (8.4%), CA—CHD (10.3%), and isolated CNS abnormalities or developmental delay (10.7%). Indications with low sample sizes (Respiratory, Other, and Musculoskeletal) were grouped together as “Other Disorders” to meet statistical assumptions. A global Fisher exact test with Monte Carlo simulation demonstrated a significant association between clinical indication and diagnostic outcome (*p* = 1 × 10^−6^), indicating that diagnostic yield differed across clinical indication categories overall. Because this analysis assessed global association across all groups simultaneously, it does not identify which specific indication categories differed from one another. Post hoc pairwise analyses were not performed, as the primary objective was to evaluate overall variation in diagnostic yield across clinical presentations.

## 4. Discussion

In this study, we evaluated the diagnostic utility of CMA among 679 critically ill neonates and children admitted to intensive care units at Phoenix Children’s Hospital between 2019 and 2024. Clinically significant copy number variants were identified in 102 (15.0%) patients, and variants of uncertain significance were observed in 139 (20.5%). Notably, the diagnostic yield of CMA is often higher in inpatient cohorts with syndromic or multisystem presentations. For example, Sanri et al. reported a diagnostic yield of 20.2% among patients with syndromic features, compared to 11.8% in those with isolated developmental delay or intellectual disability, underscoring the enhanced value of CMA in acutely ill or diagnostically complex populations [17]. These findings are consistent with prior reports of 10–20% yield in pediatric populations and reinforce CMA’s role as a first-tier diagnostic tool in the inpatient setting, especially for patients with complex phenotypes [6,7,18,19].

When considering clinical indications, the highest diagnostic yield in our study was observed in patients with multiple congenital anomalies including a congenital heart defect (CHD + CA, 48.4%). This finding is consistent with previous studies demonstrating a substantially higher diagnostic yield among patients with CHDs and other congenital anomalies compared with those with isolated CHDs [20]. Although a high diagnostic yield was also observed among patients with concurrent congenital heart defects and central nervous system abnormalities or developmental delay (CHD + CNS/DD, 53.8%), the limited number of affected individuals with pathogenic or likely pathogenic CMA findings (7 of 13) warrants cautious interpretation, and validation in larger, independent cohorts is necessary to confirm this association. These findings highlight the enhanced utility of CMA in patients with multisystem involvement and structural anomalies, where the likelihood of an underlying genomic etiology is increased. In contrast, lower yields were observed in patients with isolated findings, such as isolated congenital heart defects (CHD, 8.4%) and isolated central nervous system abnormalities or developmental delay (CNS/DD, 10.7%), which may reflect non-genomic etiologies, polygenic contributions, environmental influences, monogenic causes, or variants undetectable by CMA. Due to these reasons, ES/GS may have increased utility in isolated CHD cases when CMA is nondiagnostic. These diagnostic yields are consistent with those previously reported in cohorts with isolated CHD [21,22,23,24] and isolated DD [25].

Recurrent CNV syndromes accounted for the majority of positive findings in this cohort (n = 49, 48.0%), with deletions involving 22q11.2 being the most frequently identified (n = 27, 26.5%). This finding is consistent with prior literature characterizing 22q11.2 deletions as a leading cause of conotruncal congenital heart defects, immune dysfunction, and neurodevelopmental manifestations [26,27]. Other recurrent CNVs included 7q11.23 deletions (n = 4, 3.9%) and 16p11.2 deletions or duplications (n = 4, 3.9%), both of which are well-recognized contributors to syndromic and neurodevelopmental phenotypes [28,29,30].

Population-based and cohort studies suggest that 22q11.2 deletions account for approximately 1–5% of all congenital heart defects, with higher frequencies often in the 10–20% range and up to 20% or more for specific defects such as tetralogy of Fallot or interrupted aortic arch type B in cohorts enriched for conotruncal defects [31,32,33,34,35,36]. By contrast, studies of hospitalized infants with severe or surgically managed CHD report abnormal CMA yields of approximately 15–35%, with 22q11.2 deletions consistently among the most common recurrent pathogenic CNVs [22,37]. Collectively, these comparisons support the interpretation that critically ill ICU patients with multisystem involvement represent an enriched subgroup in whom recurrent genomic syndromes, particularly 22q11.2 deletions, are detected more frequently than in unselected outpatient CHD cohorts.

Beyond recurrent syndromes, 17 patients carried nonrecurrent CNVs overlapping genomic regions previously associated with syndromic disease. These findings underscore the challenges of establishing genotype–phenotype correlations in neonates, where variable expressivity and incomplete penetrance may obscure early recognition of classic syndromes. Furthermore, because 85% of patients in this cohort underwent testing within the first month of life, many had not yet developed distinguishing clinical features. These observations emphasize the importance of longitudinal follow-up and age-appropriate reevaluation to refine interpretation over time. This is particularly relevant for variants of uncertain significance, as ongoing phenotypic evolution, accumulation of published case data, and advances in CNV interpretation frameworks may result in future variant reclassification and refinement of genotype–phenotype comparisons. Importantly, earlier molecular diagnosis may also facilitate syndrome-specific surveillance, anticipatory management, and diagnosis-guided health monitoring before the full phenotypic spectrum becomes clinically apparent. In some cases, early genomic diagnosis may additionally help expand the recognized phenotypic spectrum associated with these syndromes.

Rare and complex genomic findings were also detected, including 22 cases of aneuploidy, 2 cases of suspected uniparental disomy (UPD14 and UPD15), and additional cases involving sex chromosome mosaicism or karyotype–phenotype discordance. The observed aneuploidy diagnostic rate of 3.2% (22/679) in our cohort is consistent with rates reported in prior CMA studies [19]. These results highlight the value of single nucleotide polymorphism (SNP) based microarray platforms in detecting alterations that may be missed by traditional karyotyping or array comparative genomic hybridization (array-CGH), such as regions of absence of heterozygosity, imprinting disorders, and chromosomal mosaicism.

Importantly, 10 patients were identified with two pathogenic or likely pathogenic CNVs. These cases often presented with complex, multisystem phenotypes, and in several instances, clinical features appeared to reflect combined effects from both CNVs. While rare, multilocus genomic variation is increasingly recognized in clinical genomic testing and underscores the need for comprehensive genomic evaluation in cases with atypical or discordant presentations [9].

Beyond establishing a genetic diagnosis, CMA findings may provide clinically actionable information relevant to ICU management. Identification of pathogenic CNVs may inform prognosis, guide syndrome-specific surveillance for associated systemic complications, support anticipatory management strategies, and facilitate subspecialty referral. In critically ill infants with complex congenital anomalies, early genomic diagnosis may also assist with surgical planning, clarify recurrence risk for families, guide genetic counseling, and support informed medical decision-making regarding escalation of care and long-term management. Additionally, recognition of specific syndromic diagnosis may help avoid unnecessary diagnostic testing and streamline inpatient evaluation.

This study has several strengths, including a large cohort size, a focus on critically ill pediatric patients, and systematic clinical-genomic correlation. However, limitations include the retrospective design, variability in phenotypic documentation, inability to follow up certain cases with confirmatory testing (e.g., CMRES-1392, CMRES-2469), and the absence of longitudinal follow-up to assess long-term outcomes. The cohort included patients from multiple intensive care settings, including the NICU, PICU, and CVICU, encompassing a broad spectrum of clinical phenotypes, illness severities, and pretest probabilities for underlying genetic disease. This heterogeneity may have influenced diagnostic yield estimates and may limit the generalizability of findings to specific ICU subpopulations or more clinically homogenous cohorts. CMA also lacks the resolution to detect single nucleotide variants or small insertions and deletions, which may explain undiagnosed cases within the cohort.

In accordance with the recent AAP recommendations, genome or exome sequencing is increasingly recommended as a first-tier diagnostic test for children with global developmental delay or intellectual disability given its superior yield and cost-effectiveness. Accordingly, there has been an increase in the implementation of rapid ES/GS programs in the NICU setting. However, despite the growing role of rapid sequencing, CMA continues to provide important diagnostic value due to its robust detection of pathogenic CNVs, aneuploidy, regions of AOH, and structural chromosomal abnormalities that may not be consistently identified in all sequencing pipelines. In many institutions, CMA remains an important complementary genomic tool that may be performed sequentially or concurrently with exome sequencing, to optimize detection of copy number and structural chromosomal abnormalities [11].

These findings support the continued integration of CMA into early diagnostic workflows in neonatal and pediatric ICU patients, particularly those with multiple congenital anomalies or complex clinical presentations. Given its high diagnostic yield and rapid turnaround time, CMA remains a valuable component of genomic evaluation workflows for critically ill neonatal and pediatric patients. Future studies incorporating longitudinal follow-up and complementary genomic technologies will be essential for enhancing diagnostic accuracy and advancing precision care in this high-risk population.

## 5. Conclusions

CMA identified clinically relevant chromosomal abnormalities in 15.0% of cases in our cohort, including recurrent and non-recurrent CNVs, common aneuploidies, and complex genomic alterations such as UPD and sex chromosome mosaicism.

Our findings reinforce the clinical utility and diagnostic value of chromosomal microarray analysis in the ICU population, particularly among neonates and infants with congenital anomalies, dysmorphic features, or unexplained critical illness.

These results underscore the continued clinical utility of CMA within contemporary genomic evaluation workflows in the ICU setting and highlight the value of early genetic evaluation to inform clinical management, prognosis, and family counseling. Notably, many patients in this cohort were tested within the first days or weeks of life, when hallmark features of syndromic conditions may not yet be evident. Accordingly, longitudinal clinical follow-up is critical for delineating the evolving phenotype and refining the interpretation of large or atypical CNVs identified during the neonatal period. Future efforts should prioritize integrating CMA with next-generation sequencing approaches to further increase diagnostic yield and uncover additional genomic contributors to complex phenotypes in this vulnerable population.

## Figures and Tables

**Table 1:** Clinically significant CNVs and relative proportions by genetic subtype.

Clinically Significant CNVs	Relative Proportions (N = 102), n (%)
Single Pathogenic or Likely Pathogenic CNV	66 (64.7%)
Recurrent Microdeletion Syndromes	41 (40.2%)
1q21.1	2 (2.0%)
7q11.23	4 (3.9%)
15q11.2	4 (3.9%)
16p11.2	3 (2.9%)
17q12	1 (1.0%)
22q11.21	27 (26.5%)
Recurrent Microduplication Syndromes	8 (7.8%)
16p11.2	1 (1.0%)
16p13.11	1 (1.0%)
17q12	1 (1.0%)
22q11.2	5 (4.9%)
Nonrecurrent Deletions	13 (12.7%)
Nonrecurrent Duplications	4 (3.9%)
Two Pathogenic/Likely Pathogenic CNVs	10 (9.8%)
Large AOH Consistent with UPD	2 (2.0%)
UPD14	1 (1.0%)
UPD15	1 (1.0%)
Aneuploidies and Others	24 (23.5%)
Trisomy 21	16 (15.7%)
Trisomy 18	3 (2.9%)
Trisomy 13	1 (1.0%)
Monosomy X	2 (2.0%)
46,X/46,XY Mosaicism	1 (1.0%)
Phenotypic Female with 46,XY Karyotype	1 (1.0%)

**Table 2:** CMA results and clinical phenotype of patients with rare or non-recurrent clinically significant CNVs.

Case Number	Age at Testing; Gender	Clinical Phenotype from Chart Review	CNV Type	Size (Kb)	Region	CNV Class	Associated with Known Syndrome
CMRES–2910	6 wk; F	Apnea, bronchiolitis, LVNC	Loss	2430	arr[GRCh37] 1p36.32 (2,628,158–5,060,971) × 1	LP	1p36 microdeletion syndrome
CMRES–2926	2 wk; M	ASD, ear malformations, hypothermia, IUGR, VSD	Loss	25,200	arr[GRCh37] 1q25.1–q32.1 (174,360,902–199,565,997) × 1	P	No
CMRES–0196	6 d; M	IUGR, oligohydramnios, GDM, elevated creatinine, hypotonia, hypospadias, inguinal hernia, low set ears, PRS, renal hypoplasia, respiratory failure	Gain	23,000	arr[GRCh37] 2p22.1–p15 (40,505,796–63,510,902) × 3	LP	No
CMRES–0255	4 d; M	oligohydramnios, DD, abnormal EEG, bilateral CHL, HRV, lung hypoplasia, pulmonary atresia	Loss	2660	arr[GRCh37] 2q37.3 (240,123,897–242,783,384) × 1	P	2q37 deletion syndrome
CMRES–1709	6 d; F	ear malformations, eye anomalies, CA, VSD, high aortic arch, aortic dilatation, thickened pulmonary valve, aortopulmonary collateral, enlarged 4th ventricle, respiratory failure, hypotonia, short stature	Loss	14,300	arr[GRCh37] 3p14.1–p12.2 (68,898,172–832,44,508) × 1	P	3p deletion syndrome
CMRES–1665	2 wk; F	DD, epilepsy, cerebral infarction due to left MCA embolism, hemiplegia, hemiparesis, HLHS, VSD, PDA, PH, bilateral CHL, feeding difficulty, GERD	Loss	5000	arr[GRCh37] 4q31.21–q31.22 (143,381,651–148,378,867) × 1	LP	No
CMRES–0225	2 d; M	ASD, GI/GU abnormalities, imperforate anus	Gain	16,500	arr[GRCh37] 8q12.1–q21.11 (60,765,051–77,234,448) × 3	P	8q duplication syndrome
CMRES–2505	3 wk; M	DD, feeding difficulties, ASD, PFO, dysmorphic facial features, brachycephaly	Gain	39,631	arr[GRCh37] 8q21.2–q24.13 (85,850,967–125,482,371) × 3	P	No
CMRES–0040	1 yr; M	epilepsy, GDD, OSA, feeding difficulties, tracheomalacia, FTT, abnormal head movements, dysmorphic facial features, ear malformations, bilateral CHL, NTD, congenital sacral dimple	Gain	25,300	arr[GRCh37] 9p24.3–p21.3 (203,861–25,537,584) × 3	P	9p duplication syndrome
CMRES–3583	2 wk; F	macrosomia, limb anomaly, COA, omphalocele	Loss	16,600	arr[GRCh37] 9p24.3–p22.2 (203,862–16,767,774) × 1	P	9p deletion syndrome
CMRES–1314	4 d; F	DD, feeding difficulties, COA, BAV, HLV, AKI, LA, elevated LFTs, possible seizure activity, abnormal brain MRI	Loss	179	arr[GRCh37] 9q34.3 (139,217,461–139,396,216) × 1	LP	9q34.3 deletion
CMRES–1376	4 d; M	HLHS	Loss	85	arr[GRCh37] 9q34.3 (139,341,866–139,427,066) × 1	P	NOTCH1–related condition
CMRES–1344	6 d; M	DD, feeding difficulties, ear malformations, COA, PDA, VSD, respiratory failure	Loss	1110	arr[GRCh37] 12q–15q21.1 (70,403,305–71,510,598) × 1	LP	12q15 microdeletion syndrome
CMRES–1019	9 wk; M	IUGR, FTT, dysmorphic facial features, bilateral retinoblastoma	Loss	38,000	arr[GRCh37] 13q12.3–q21.33 (30,706,913–68,730,584) × 1	P	13q deletion syndrome
CMRES–0678	2 wk; M	GDM, IUGR, respiratory distress, focal seizures, COA, microcephaly, dysmorphic facial features, abnormal EEG, hypotonia, GERD, coloboma	Loss	5560	arr[GRCh37] 14q32.2–q32.33 (100,765,047–106,329,074) × 1	P	14q32 imprinting disorder (Temple vs. Kagami–Ogata), origin not established
CMRES–1001	3 mo; M	TOF, PAS, hepatic hemangioma	Loss	6050	arr[GRCh37] 18p11.32–p11.31 (136,226–6,185,609) × 1	P	18p deletion syndrome
CMRES–1698	4 d; F	TAPVR, structural brain anomaly	Loss	8430	arr[GRCh37] 22q13.2–q13.33 (42,755,628–51,183,872) × 1	P	Phelan–McDermid syndrome

Abbreviations: d, day; wk, week; mo, month; yr, year; F, female; M, male; LP, likely pathogenic; P, pathogenic; AKI, acute kidney injury; ASD, atrial septal defect; BAV, bicuspid aortic valve; CA, choanal atresia; CHL, conductive hearing loss; COA, coarctation of aorta; DD, developmental delay; EEG, electroencephalogram; FTT, failure to thrive; GDD, global developmental delay; GDM, gestational diabetes mellitus; GI/GU, gastrointestinal/genitourinary; HLHS, hypoplastic left heart syndrome; HLV, hypoplastic left ventricle; HRV, hypoplastic right ventricle; IUGR, intrauterine growth restriction; LA, lactic acidosis; LFT, liver function test; LVNC, left ventricular non-compaction; MCA, middle cerebral artery; NTD, neural tube defect; OSA, obstructive sleep apnea; PAS, pulmonary artery stenosis; PDA, patent ductus arteriosus; PH, pulmonary hypertension; PFO, patent foramen ovale; PRS, Pierre Robin sequence; TAPVR, total anomalous pulmonary venous return; TOF, tetralogy of Fallot; VSD, ventricular septal defect.

**Table 3:** CMA results and clinical phenotype of patients with two pathogenic or likely pathogenic CNVs.

Case Number	Age; Gender	Clinical Phenotype	CNV Type	Size (kb)	Region	CNV Class
CMRES-1947	6 d; F	coronary artery fistula, respiratory failure, metabolic acidosis, suspected seizure, hydrocephalus, dysmorphic features, ventriculomegaly, lissencephaly, polymicrogyria, PH, CP	Loss	4420	arr[GRCh37] 1p36.33–p36.32 (849,466–5,264,535) × 1	P
			Gain	18,600	arr[GRCh37] 14q31.3–q32.33 (88,703,186–107,285,437) × 3	P
CMRES-1392	7 d; M	polyhydramnios, hypertonia, ear malformations, facial cleft, CHL, right hemifacial microsomia	Gain	11,800	arr[GRCh37] 2q33.1–q34 (202,035,063–213,827,186) × 3	P
			Gain	8120	arr[GRCh37] 17p11.2–q11.2 (19,257,545–27,378,279) × 3	P
CMRES-1388	2 wk; M	IUGR, respiratory distress, ASD, congenital malformations of ribs, craniosynostosis, hemivertebrae, dysmorphic features, congenital ptosis, mandibular hypoplasia, short palpebral fissures, hypoplastic anterior fontanelle, sacral dimple, low set ears, feeding difficulties, FTT, scoliosis	Loss	3580	arr[GRCh37] 2q37.3 (239,203,172–242,783,384) × 1	P
			Gain	30,300	arr[GRCh37] 6p25.3–p21.33 (156,974–30,487,974) × 3	P
CMRES-2469	3 wk; F	IUGR, FTT, laryngomalacia/stridor, respiratory distress	Loss	5610	arr[GRCh37] 5p15.33–p15.31 (113,577–5,723,670) × 1	P
			Loss	10,300	arr[GRCh37] 5p15.33–p15.31 (5,771,241–15,984,451) × 1~2	P
CMRES-0763	6 d; F	HLHS, hypoplastic aortic arch, anterior anus, coloboma, cranial deformity, choanal atresia, thrombocytopenia, PH	Gain	7170	arr[GRCh37] 5p15.33–p15.31 (113,576–7,283,299) × 3	P
			Loss	15,000	arr[GRCh37] 11q23.3–q25 (119,975,949–134,938,470) × 1	P
CMRES-1864	4 d; F	TOF, agenesis of corpus callosum, absent septum pellucidum, optic nerve hypoplasia, feeding difficulties	Loss	1830	arr[GRCh37] 6q27 (169,085,221–170,919,482) × 1	P
			Gain	17,800	arr[GRCh37] 21q21.3–q22.3 (30,261,221–48,097,372) × 3	P
CMRES-0834	5 wk; M	dysmorphic facial features, hypotonia, deep creases separating 1st toes bilaterally, inguinal hernia, respiratory distress, FTT, hypotonia, tracheomalacia, hearing loss, DD, multiple congenital malformations, diffuculty feeding, constipation	Loss	6890	arr[GRCh37] 8p23.3–p23.1 (158,048–7,044,046) × 1	P
			Gain	31,300	arr[GRCh37] 8p23.1–p11.1 (12,528,482–43,786,723) × 3	P
CMRES-2183	13 d; F	truncus arteriosus, bilateral PA hypoplasia, renal dysplasia, growth retardation, microcephaly, DD	Loss	10,400	arr[GRCh37] 9q21.11–q21.13 (68,734,572–79,156,769) × 1	P
			Loss	2550	arr[GRCh37] 22q11.21 (18,916,843–21,465,659) × 1	P
CMRES-1150	5 d; F	HLV, VSD, interrupted aortic arch, hypoplasia of corpus callosum, focal epilepsy, DD	Gain	24,000	arr[GRCh37] 12q24.11–q24.33 (109,732,471–133,777,902) × 3	P
			Loss	4360	arr[GRCh37] 11q24.3–q25 (130,574,610–134,938,470) × 1	LP
CMRES-3688	4 d; M	TOF, CP, dysmorphic facial features, ear malformations	Gain	25,900	arr[GRCh37] 14q11.2–q21.2 (20,511,673–46,421,909) × 3	P
			Gain	29,700	arr[GRCh37] 3p26.3–p24.1 (61,892–29,803,397) × 3	P

Abbreviations: d, day; wk, week; mo, month; yr, year; F, female; M, male; LP, likely pathogenic; P, pathogenic; ASD, atrial septal defect; CHL, conductive hearing loss; CP, cleft palate; DD, developmental delay; FTT, failure to thrive; HLHS, hypoplastic left heart syndrome; HLV, hypoplastic left ventricle; IUGR, intrauterine growth restriction; PA, pulmonary artery; PH, pulmonary hypertension; TOF, tetralogy of Fallot; VSD, ventricular septal defect.

**Table 4:** Diagnostic yield of chromosomal microarray (CMA) by primary clinical indication at time of referral.

Clinical Indications at Time of Referral	ICU Cases and Relative Proportions (N = 679), n (%)	Clinically Significant CNVs and Relative Proportions (N = 102), n (%)	Detection Rate for Clinical Indication, % (95% CI)
CHD + others	501 (73.8)	85 (83.3)	17% (13.9%–20.5%)
CHD (isolated)	395 (58.2)	33 (32.4)	8.4% (6.0%–11.5%)
CHD + CNS/DD	13 (1.9)	7 (6.8)	53.8% (29.1%–76.8%)
CHD + CA	93 (13.7)	45 (44.1)	48.4% (38.5%–58.4%)
CA—CHD	117 (17.2)	12 (11.8)	10.3% (6.0%–17.1%)
CNS/DD (isolated)	28 (4.1)	3 (2.9)	10.7% (3.7%–27.2%)
Other	33 (4.9)	2 (2.0)	6.1% (1.7%–19.6%)

## Data Availability

All shareable data from patients are presented in the manuscript. De-identified case-level data are not available for sharing under the terms of the IRB approval.
